# Comparative efficacy of different treatments for menstrual migraine: a systematic review and network meta-analysis

**DOI:** 10.1186/s10194-023-01625-x

**Published:** 2023-07-03

**Authors:** Han Zhang, Jian-Zhi Qi, Zhi-Hua Zhang

**Affiliations:** 1 The Second School of Clinical Medicine, Anhui Medical University, Hefei, 230032 China; 2 Department of Epidemiology and Biostatistics, School of Public Health, Anhui Medical University, Hefei, 230032 China; 3 Center for Evidence Based Medicine, Anhui Medical University, Hefei, 230032 China

**Keywords:** Menstrual migraine, Migraine disease, Randomized controlled trials, Network meta-analysis, Systematic review

## Abstract

**Background:**

Menstrual migraine is a subtype of migraine disease that is typically more disabling, longer-lasting, and more challenging to treat. The purpose of this network meta-analysis (NMA) is to compare the relative efficacy of treatments for menstrual migraine.

**Methods:**

We systematically searched databases, including PubMed, EMBASE, and Cochrane, and included all eligible randomized controlled trials in the study. We conducted the statistical analysis using Stata version 14.0, based on the frequentist framework. We used the Cochrane Risk of Bias tool for randomized trials version 2 (RoB2) to assess the risk of bias of the included studies.

**Results:**

This network meta-analysis included 14 randomized controlled trials with 4601 patients. For short-term prophylaxis, frovatriptan 2.5 mg twice daily had the highest probability of effectiveness [OR = 1.87 (95% CI: 1.48 to 2.38)] compared to placebo. For acute treatment, the results showed that sumatriptan 100 mg [OR = 4.32 (95% CI: 2.95 to 6.34)] was the most effective treatment compared to placebo.

**Conclusions:**

These findings suggest that frovatriptan 2.5 mg twice daily was best for short-term prevention, sumatriptan 100 mg were best for acute treatment. More high-quality randomized trials are required to determine the most effective treatment.

**Supplementary Information:**

The online version contains supplementary material available at 10.1186/s10194-023-01625-x.

## Introduction

Migraine is a common neurological disorder that affects women more than men. One specific type of migraine that targets women is menstrual migraine, which occurs exclusively or mainly during the hormonal changes before or during menstruation. The diagnostic criteria for menstrual migraine are placed in the appendix of the International Classification of Headache Disorders third edition (ICHD-3) as research criteria that need validation [1]. According to ICHD-3, menstrual migraine can be divided into two types: pure menstrual migraine (PMM) and menstrually related migraine (MRM) (Table 1).

**Table 1:** Diagnostic criteria

pure menstrual migraine (PMM)
Patients must fit ICHD-3 criteria for migraine and have migraine attacks occurring only during a 5-day menstrual period (days − 2 to + 3 of menstruation) in at least two of every three menstrual cycles and at no other times of the cycle
menstrually related migraine (MRM)
Patients must fit ICHD-3 criteria for migraine and have migraine attacks in a 5-day menstrual period (days − 2 to + 3 of menstruation) in at least two of every three menstrual cycles and additionally at other times of the month outside the cycle

Previous studies have reported that 18–25% of female migraine sufferers experience menstrual migraine without aura [2]. Compared to nonmenstrual attacks, menstrual migraine attacks tend to be more severe, longer-lasting and less responsive to treatment, resulting in a significant reduction of the quality of life for the affected women [3, 4]. Several medications, especially triptans such as sumatriptan and frovatriptan, have demonstrated efficacy for the acute treatment and short-term prophylaxis of menstrual migraine.

However, the relative efficacy of these medications are difficult to compare due to the lack of head-to-head studies among them. Only one meta-analysis has assessed the effects of different drugs on the short-term prophylaxis of menstrual migraine, while no studies have analyzed and evaluated the acute treatment [5]. Other narrative reviews have only summarized the various treatment options, without providing clear recommendations for treatment priority [2, 6–9]. This poses a challenge for clinicians to select appropriate treatments. To address this gap, we conducted a network meta-analysis (NMA) to systematically evaluate the efficacy of various interventions for menstrual migraine based on direct and indirect evidence from randomized controlled trials (RCTs).

## Methods

### Search strategy

The present systematic review and meta-analysis is reported according to the Preferred Reporting Items of Systematic Reviews and Meta-Analyses (PRISMA) extension statement [10]. We conducted a comprehensive search of PubMed, Embase, and Cochrane databases from their inception until September 2022, with an updated search performed in March 2023. The detailed search strategy is provided in the supplement eTable 1. The review protocol was prospectively registered on PROSPERO (CRD42022329011).

### Study selection

In this article, we divided treatment options for menstrual migraine into three categories: long-term prophylaxis, short-term prophylaxis, and acute treatment. To be included in our study, studies needed to meet the following PICOS criteria.

- (1) Population(P): We diagnosed participants with menstrual migraine according to ICHD-3 or criteria that closely matched ICHD-3. Specifically, migraine attacks occurred on days -2 to + 3 (or days -2 to + 4) of menstruation in at least two out of three menstrual cycles (2/3-criterion). However, since there were no clear diagnostic criteria for menstrual migraine before ICHD-2, we also included some alternative criteria: migraine attacks occurred in at least three out of four menstrual cycles (3/4-criterion); migraine attacks occurred in the last menstrual cycle (1/1-criterion).
- (2) Intervention(I): We included relevant interventions, such as triptans, estrogen supplementation, etc. We did not restrict the types of interventions, but not all of them could be included in the data analysis due to different trial designs and outcome measures. For some interventions that could not be statistically analyzed, we provided a narrative summary.
- (3) Comparison(C): Studies contained comparisons between different treatments;
- (4) Outcome (O): At least one of the following outcomes: mean percentage of perimenstrual periods (PMPs) without menstrual-related migraine (MRM), 2-h pain freedom (percentage of patients or attacks).
- (5) Study design (S): Randomized clinical trials (RCTs).

### Quality assessment

We assessed the quality of each trial using version 2 of the Cochrane risk-of-bias tool for RCTs [11]. Two investigators (H.Z. and J.-Z.Q.) independently performed the assessment and any disagreements were resolved by consensus.

### Outcomes and data Collection

Before conducting formal data extraction, we summarized all available endpoints and identified those that were suitable for NMA. For the assessment of short-term prophylaxis, we selected “Mean percentage of PMPs without MRM” as the primary efficacy outcome and “any adverse events” as the secondary safety outcome. For acute treatment assessment, we chose “2-h pain freedom (percentage of patients)” as the primary efficacy outcome, “2-h pain freedom (percentage of attacks)”, “2–24 h sustained pain freedom (percentage of patients)”, and “Recurrent episodes at 24 h (percentage of attacks)” as the secondary efficacy outcomes. To ensure data accuracy, two investigators (H.Z. and J.-Z.Q.) independently extracted the data. If relevant data were missing in the studies, we contacted the authors or co-authors to obtain the original data.

### Data analysis

Using Stata, version 14.0, we performed a network meta-analysis based on the frequentist framework that synthesized direct and indirect evidence from trials comparing different treatments (including multi-arm trials). We assumed random effects models for all analyses and summarized effect sizes for dichotomous variables using odds ratio (OR) with corresponding 95% confidence intervals (CIs). We assessed heterogeneity graphically for pair-wise comparisons and reported the I2 index and p-values for the Cochrane Q test. We evaluated local inconsistency by using the nodesplit approach. To visualize network geometry and node connectivity, we produced network evidence plots for each outcome. To determine the relative merits of different treatments, we used a ranking approach based on the surface under the cumulative ranking curve (SUCRA), which ranges from 0 to 100%, with higher values indicating higher ranks. We performed a sensitivity analysis, excluding studies in which migraine occurred on days -2 to + 4 of the menstrual cycle, and again combining effect sizes for the primary outcome of the remaining studies. To explore the possibility of publication bias, we constructed funnel plots for the primary outcomes and visually inspected them for asymmetry.

## Results

### Study characteristics

We conducted a literature search and identified 1493 articles, of which we screened 60 full-text articles for eligibility. As eFigure 1 shows, we excluded 39 articles for various reasons. We updated the search in March 2023 and found no articles. Thus, we included 14 studies for qualitative synthesis [12–25]. Table 2 summarizes the characteristics of these studies. eFigure 2 displays the risk of bias judgments for the studies contributing to the analysis of each outcome. We found that 7% (1/14 items), 93% (13/14 items), and 0% (0/19 items) of the included studies had low, some concerns, and high risk of bias, respectively. No loops of evidence allowed for an assessment of inconsistency. eFigure 3 presents the pairwise analyses and heterogeneity test results. We only detected substantial heterogeneity in the pairwise comparison of sumatriptan–naproxen with placebo for recurrent episodes at 24 h (I^2^ = 77.4%, *P* = 0.036).

**Table 2:** Characteristics of the included studies

Study	Total No.of patients	Diagnostic criteria	Intervention	Control	Outcomes
**Short-term prophylaxis**					
Silberstein SD,2004	506	3/4-criterion,-2 to + 4 days	Frovatriptan 2.5 mg QD/BID from day -4 to day + 2	Placebo from day -4 to day + 2	①②
Brandes JL,2009	410	2/3-criterion,-2 to + 3 days	Frovatriptan 2.5 mg QD/BID from day -2 to day + 4	Placebo from day -2 to day + 4	①②
Newman L,2001	136	1/1-criterion,-2 to + 3 days	Naratriptan 1 mg BID from day -2 to day + 3	Placebo from day -2 to day + 3	①②
Mannix LK,2007	633	1/1-criterion,-2 to + 4 days	Naratriptan 1 mg BID from day -3 to day + 3	Placebo from day -3 to day + 3	①②
**Acute treatment**					
Mannix LK,2009	621	2/3-criterion,-2 to + 3 days	Sumatriptan–naproxen	Placebo	③④
Martin V,2008	94	2/3-criterion,-2 to + 3 days	Rizatriptan 10 mg	Placebo	③④
Nett R,2008	705	2/3-criterion,-2 to + 3 days	Rizatriptan 10 mg	Placebo	③④
Massiou H,2005	229	2/3-criterion,-2 to + 4 days	Naratriptan 2.5 mg	Placebo	③
Allais G,2011(Cephalalgia)	244	2/3-criterion,-2 to + 3 days	Almotriptan 12.5 mg	Placebo	③④
Landy S,2004	752	2/3-criterion,-2 to + 4 days	Sumatriptan 50 mg/100 mg	Placebo	③④
Bartolini M,2012	67	2/3-criterion,-2 to + 3 days	Almotriptan 12.5 mg	Frovatriptan 2.5 mg	⑤⑥
Allais G,2011(Neurol Sci)	76	2/3-criterion,-2 to + 3 days	Zolmitriptan 2.5 mg	Frovatriptan 2.5 mg	⑤⑥
Savi L,2011	93	2/3-criterion,-2 to + 3 days	Rizatriptan 10 mg	Frovatriptan 2.5 mg	⑤⑥
Bigal M,2008	35	2/3-criterion,-2 to + 3 days	Rizatriptan 10 mg	Dexamethasone 4 mg	⑤

Outcomes: ① Mean percentage of PMPs without MRM; ② All adverse events; ③ 2-h pain freedom (percentage of patients); ④ 2–24 h sustained pain freedom(percentage of patients); ⑤ 2-h pain freedom (percentage of attacks); ⑥ Recurrent episodes at 24 h (percentage of attacks);

Abbreviations: *MM *Menstrual migraine, *PMM *Pure menstrual migraine, *MRM *Menstrually related migraine, MM* Difficult-to-treat menstrual migraine, *QD *Once a day, *BID *Twice a day

## Outcomes

### Mean percentage of PMPs without MRM and all adverse events

We compared the efficacy and safety of different interventions for short-term prophylaxis. Four articles with four individual treatment arms were included in the NMA (eFigure 5A). All interventions had a significantly higher mean percentage of PMPs without MRM compared to the placebo, including naratriptan 1 mg BID [OR = 1.75 (95% CI: 1.29 to 2.38)], frovatriptan 2.5 mg QD [OR = 1.87 (95% CI: 1.48 to 2.38)], and frovatriptan 2.5 mg BID [OR = 2.80 (95% CI: 2.20 to 3.57)] (Table 3A). The SUCRA results indicated that frovatriptan 2.5 mg BID achieved the highest improvement in mean percentage of PMPs without MRM among all interventions (eTable 2A). For safety outcome, none of the medications had significantly fewer adverse events than the placebo (Table 3B). Based on the SUCRA, frovatriptan 2.5 mg BID was associated with the least adverse events (eTable 2B).

**Table 3:** League table of pairwise comparisons in network meta-analysis, expressed as OR [95% CI]

**A.** Mean percentage of PMPs without MRM			
Placebo	1.87 (1.48,2.38)	2.80 (2.20,3.57)	1.75 (1.29,2.38)
0.53 (0.42,0.68)	Frovatriptan 2.5 mg QD	1.50 (1.19,1.89)	0.93 (0.63,1.38)
0.36 (0.28,0.45)	0.67 (0.53,0.84)	Frovatriptan 2.5 mg BID	0.62 (0.42,0.92)
0.57 (0.42,0.78)	1.07 (0.73,1.58)	1.60 (1.08,2.37)	Naratriptan 1 mg BID
**B.** All adverse events			
Placebo	1.06 (0.71,1.58)	0.94 (0.61,1.45)	1.58 (1.05,2.36)
0.94 (0.63,1.40)	Frovatriptan 2.5 mg QD	0.89 (0.58,1.35)	1.48 (0.84,2.62)
1.06 (0.69,1.63)	1.13 (0.74,1.72)	Frovatriptan 2.5 mg BID	1.67 (0.92,3.03)
0.63 (0.42,0.95)	0.67 (0.38,1.19)	0.60 (0.33,1.08)	Naratriptan 1 mg BID

### Primary outcome: 2-h pain freedom (percentage of patients)

**Fig. 1:**
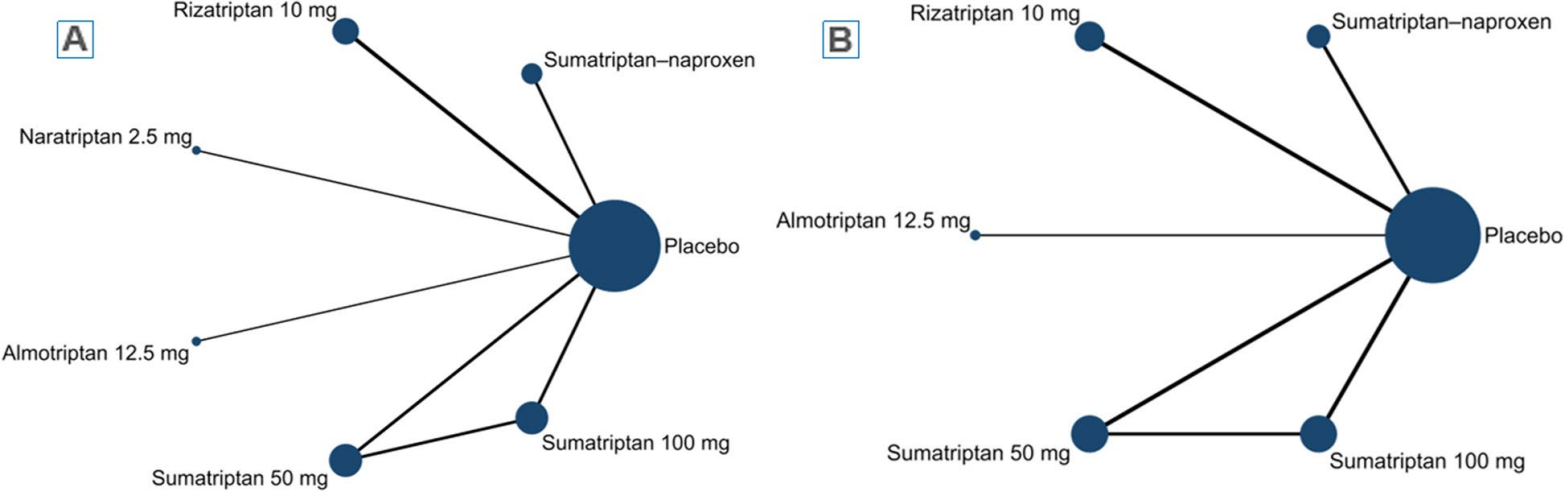
network evidence plot of (**A**) 2-h pain freedom (percentage of patients) and (**B**) 2–24 h sustained pain freedom(percentage of patients)

**Fig. 2:**
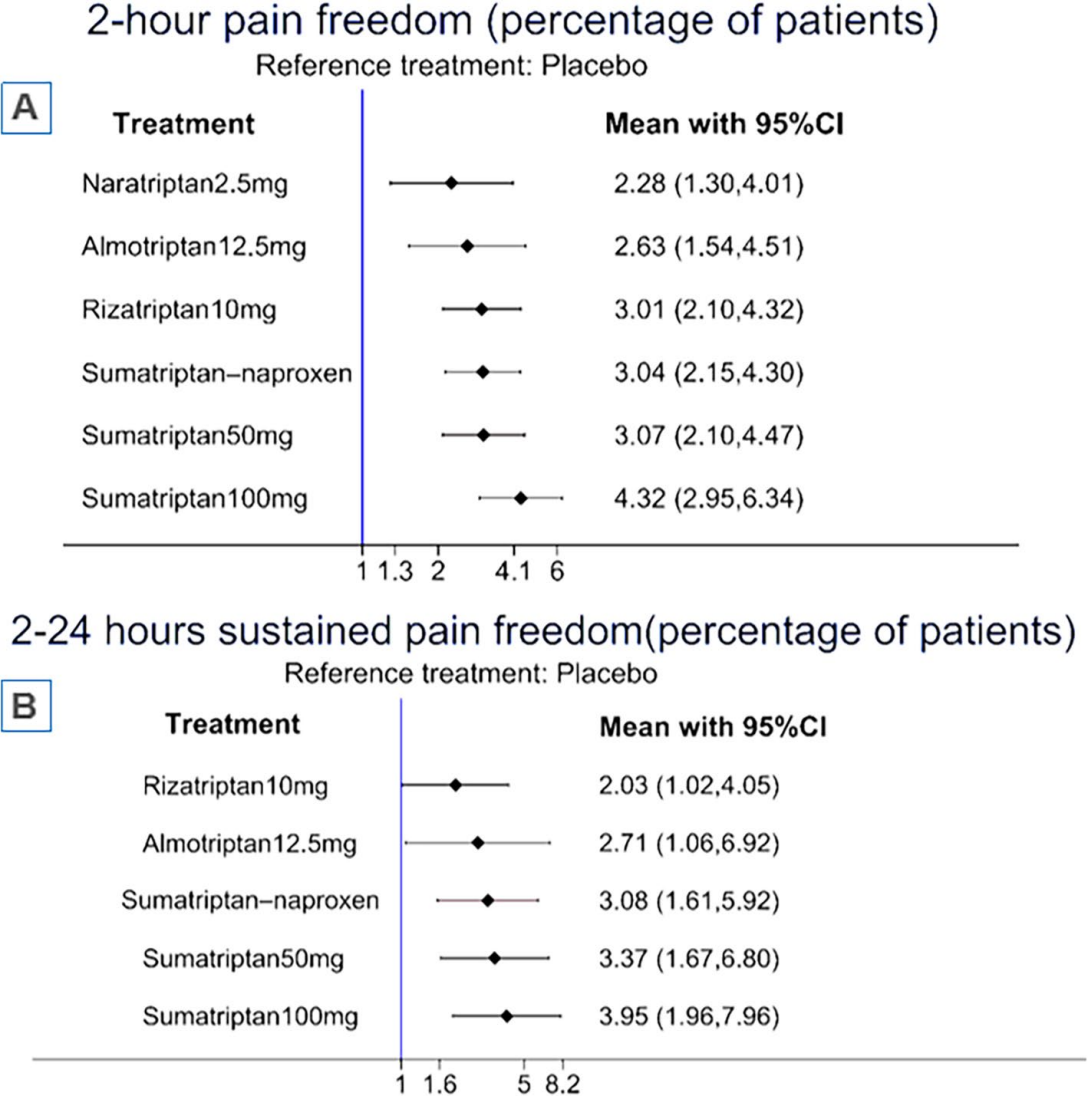
Forest plot of (**A**) 2-h pain freedom (percentage of patients) and (**B**) 2–24 h sustained pain freedom(percentage of patients)

Eight articles with eleven individual treatment arms were included in the NMA (Fig. 1A). The league table and forest plot revealed that most interventions were associated with a significantly higher OR for 2-h pain freedom (percentage of patients) compared with placebo, including naratriptan 2.5 mg [OR = 2.28 (95% CI: 1.30 to 4.01)], almotriptan 12.5 mg [OR = 2.63 (95%CI: 1.54 to 4.51)], rizatriptan 10 mg [OR = 3.01 (95%CI: 2.10 to 4.32)], sumatriptan–naproxen [OR = 3.04 (95%CI: 2.15 to 4.30)], sumatriptan 50 mg [OR = 3.07 (95% CI: 2.10 to 4.47)], sumatriptan 100 mg [OR = 4.32 (95% CI: 2.95 to 6.34)] (Fig. 2A, Table 4A). According to the SUCRA, sumatriptan 100 mg were associated with the highest probability of effectiveness on 2-h pain freedom (percentage of patients) among all interventions (eTable 2C).

**Table 4:** League table of pairwise comparisons in network meta-analysis, expressed as OR [95% CI]

**A:** 2-h pain freedom (percentage of patients)						
Placebo	3.04 (2.15,4.30)	3.01 (2.10,4.32)	2.28 (1.30,4.01)	2.63 (1.54,4.51)	3.07 (2.10,4.47)	4.32 (2.95,6.34)
0.33 (0.23,0.47)	Sumatriptan–naproxen	0.99 (0.60,1.64)	0.75 (0.39,1.46)	0.87 (0.46,1.64)	1.01 (0.60,1.69)	1.42 (0.85,2.39)
0.33 (0.23,0.48)	1.01 (0.61,1.66)	Rizatriptan 10 mg	0.76 (0.39,1.48)	0.87 (0.46,1.67)	1.02 (0.60,1.72)	1.43 (0.85,2.43)
0.44 (0.25,0.77)	1.33 (0.69,2.58)	1.32 (0.68,2.58)	Naratriptan 2.5 mg	1.16 (0.53,2.52)	1.34 (0.68,2.65)	1.89 (0.96,3.75)
0.38 (0.22,0.65)	1.15 (0.61,2.19)	1.14 (0.60,2.19)	0.87 (0.40,1.89)	Almotriptan 12.5 mg	1.16 (0.60,2.24)	1.64 (0.85,3.17)
0.33 (0.22,0.48)	0.99 (0.59,1.66)	0.98 (0.58,1.66)	0.74 (0.38,1.47)	0.86 (0.45,1.66)	Sumatriptan 50 mg	1.41 (0.99,2.01)
0.23 (0.16,0.34)	0.70 (0.42,1.18)	0.70 (0.41,1.18)	0.53 (0.27,1.04)	0.61 (0.32,1.18)	0.71 (0.50,1.01)	Sumatriptan 100 mg
**B:** 2–24 h sustained pain freedom(percentage of patients)						
Placebo	3.08 (1.61,5.92)	2.03 (1.02,4.05)	2.71 (1.06,6.92)	3.37 (1.67,6.80)	3.95 (1.96,7.96)	
0.32 (0.17,0.62)	Sumatriptan–naproxen	0.66 (0.26,1.70)	0.88 (0.28,2.75)	1.09 (0.42,2.84)	1.28 (0.49,3.33)	
0.49 (0.25,0.98)	1.52 (0.59,3.91)	Rizatriptan 10 mg	1.33 (0.42,4.26)	1.66 (0.62,4.43)	1.94 (0.73,5.19)	
0.37 (0.14,0.94)	1.14 (0.36,3.56)	0.75 (0.23,2.40)	Almotriptan12.5 mg	1.24 (0.39,4.00)	1.46 (0.45,4.69)	
0.30 (0.15,0.60)	0.92 (0.35,2.38)	0.60 (0.23,1.61)	0.81 (0.25,2.59)	Sumatriptan50mg	1.17 (0.62,2.21)	
0.25 (0.13,0.51)	0.78 (0.30,2.03)	0.51 (0.19,1.37)	0.69 (0.21,2.21)	0.85 (0.45,1.61)	Sumatriptan100mg	

### Secondary outcome: 2–24 h sustained pain freedom(percentage of patients)

Six articles with seven different treatment arms were included in the NMA (Fig. 1B). Most interventions had a significantly higher OR for 2–24 h sustained pain freedom (percentage of patients) compared with placebo, including almotriptan 12.5 mg [OR = 2.71 (95%CI: 1.06 to 6.92)], rizatriptan 10 mg [OR = 2.03 (95%CI: 1.02 to 4.05)], sumatriptan–naproxen [OR = 3.08 (95%CI: 1.61 to 5.92)], sumatriptan 50 mg [OR = 3.37 (95% CI: 1.67 to 6.80)], sumatriptan 100 mg [OR = 3.95 (95% CI: 1.96 to 7.96)] (Fig. 2B, Table 4B). The SUCRA found that across all treatments, sumatriptan 100 mg had the highest probability of effectiveness on 2–24 h sustained pain freedom (percentage of patients) (eTable 2D).

### Secondary outcome: 2-h pain freedom (percentage of attacks)

Four articles with five individual treatment arms were investigated in the current NMA (eFigure 5B). Compared to frovatriptan 2.5 mg, none of the interventions had a significantly higher effectiveness on 2-h pain freedom (percentage of attacks) (Table 5A). According to the SUCRA, almotriptan 12.5 mg were associated with the highest probability of effectiveness on 2-h pain freedom (percentage of attacks) among all interventions (eTable 2E).

### Secondary outcome: recurrent episodes at 24 h (percentage of attacks)

Only 3 studies reported this outcome (eFigure 5C). Frovatriptan 2.5 mg had a significantly lower recurrence rate at 24 h (percentage of attacks) than almotriptan 12.5 mg and rizatriptan 10 mg, with odds ratios (ORs) of 0.33 (95% CI: 0.12 to 0.89) and 0.24 (95% CI: 0.08 to 0.70), respectively (Table 5B). According to the SUCRA results, frovatriptan 2.5 mg was the most effective intervention for reducing the recurrence rate at 24 h (percentage of attacks) among all interventions (eTable 2F).

**Table 5:** League table of pairwise comparisons in network meta-analysis, expressed as OR [95% CI]

**A.** 2-h pain freedom (percentage of attacks)				
Rizatriptan 10 mg	1.49 (0.49,4.48)	1.09 (0.35,3.36)	0.86 (0.38,1.94)	0.24 (0.11,0.52)
0.67 (0.22,2.02)	Almotriptan 12.5 mg	0.73 (0.25,2.15)	0.58 (0.27,1.22)	0.16 (0.04,0.62)
0.92 (0.30,2.85)	1.37 (0.46,4.04)	Zolmitriptan 2.5 mg	0.79 (0.36,1.73)	0.22 (0.06,0.87)
1.16 (0.52,2.62)	1.73 (0.82,3.64)	1.26 (0.58,2.76)	Frovatriptan 2.5 mg	0.28 (0.09,0.86)
4.22 (1.91,9.32)	6.27 (1.61,24.38)	4.58 (1.15,18.18)	3.63 (1.17,11.29)	Dexamethasone 4 mg
**B**. Recurrent episodes at 24 h (percentage of attacks)				
Rizatriptan 10 mg	0.73 (0.17,3.17)	0.37 (0.09,1.48)	0.24 (0.08,0.70)	
1.37 (0.32,5.93)	Almotriptan 12.5 mg	0.51 (0.13,1.91)	0.33 (0.12,0.89)	
2.70 (0.68,10.78)	1.97 (0.52,7.43)	Zolmitriptan 2.5 mg	0.65 (0.27,1.55)	
4.18 (1.43,12.24)	3.05 (1.13,8.29)	1.55 (0.65,3.70)	Frovatriptan 2.5 mg	

### Sensitivity analysis

For short-term prophylaxis, according to the sensitivity analysis done in the two remaining trials, naratriptan 1 mg BID [OR = 2.88 (95% CI: 1.40 to 5.94), SUCRA = 82.1] has a better efficacy than frovatriptan 2.5 mg BID [OR = 2.41 (95% CI: 1.29 to 4.50), SUCRA = 72.1], which is different from the overall NMA results (eFigure 6A, eTable 3A).

For acute treatment, the estimates of treatment effects in the sensitivity analysis showed only small changes compared with those in the whole network meta-analysis (eFigure 6B, eTable 3B).

### Publication bias

We performed funnel plot analyses for the primary outcomes, despite the limited number of studies (less than ten) for each outcome (eFigure 4). The funnel plot for the outcome of “Mean percentage of PMPs without MRM”exhibited some asymmetry, indicating a possible risk of publication bias.

## Discussion

This study provides suggestive evidence for the optimal treatment strategy of menstrual migraine. It indicates that frovatriptan 2.5 mg BID is the most suitable short-term prophylactic agent and sumatriptan 100 mg is the most efficacious acute treatment. In addition, we observed some discrepancies in the pharmacological response between menstrual migraine and common migraine, implying that the choice of drugs may differ somewhat.

For short-term prophylaxis, our analysis suggested that frovatriptan 2.5 mg BID might have the highest efficacy in reducing migraine days per perimenstrual period. This might be attributed to its longer half-life, which enables a more sustained effect. No serious drug-related adverse events were reported in any of the studies. Furthermore, our analysis also implied that frovatriptan 2.5 mg BID had fewer adverse events and a better safety profile. However, it is important to note that the sensitivity analysis for the primary efficacy outcome was not consistent with the results of the whole NMA analysis, and publication bias exists, requiring caution in the interpretation of the results. This difference in results might stem from the deviation of diagnostic criteria, medication duration and number of patients included. Therefore, more rigorous and standardized clinical trials are necessary in the future.

In addition to short-term prevention, long-term prophylaxis with agents such as estrogen, erenumab, etc., is also a viable option for menstrual migraine [6, 7, 9]. However, the evidence for long-term prophylaxis is still inadequate. One approach to prevent menstrual migraine is to supplement estrogen to counteract the drop that triggers migraine. Yet, most of the relevant studies are low-quality non-RCTs that were excluded from our data analysis. Moreover, some studies have shown that estradiol is effective for menstrual migraine, but the frequency of migraine increases when estrogen is discontinued. Another intervention is once-monthly subcutaneous injections of erenumab, which were also effective for menstrual migraine, but comparisons with other interventions are lacking. Therefore, we recommend short-term prevention when patients can predict their menstrual cycle, as it is supported by stronger research evidence.

For acute treatment, our analysis showed that sumatriptan 100 mg was superior to all other treatments in terms of 2-h pain freedom. However, it should be noted that this advantage was not significant compared to other interventions except placebo. Moreover, these results for menstrual migraine differ from those of common migraine. A previous NMA for common migraine showed that rizatriptan 10 mg had a better effect than sumatriptan 100 mg and 50 mg on 2-h pain freedom [26]. However, our analysis for menstrual migraine showed that sumatriptan 100 mg and 50 mg were superior to rizatriptan 10 mg. This discrepancy suggests that there may be some potential differences in drug selection between menstrual migraine and common migraine. Meanwhile, our study also showed that frovatriptan 2.5 mg had the lowest 24-h recurrence rate. Therefore, frovatriptan 2.5 mg is a good choice for menstrual migraine patients with frequent headache recurrence, although it is less effective than other drugs in achieving 2-h pain freedom. Due to the scarcity of data, we did not conduct a safety analysis of the drugs used for acute treatment of menstrual migraine. However, rizatriptan, naratriptan and almotriptan all showed good safety profiles [17–19, 27].

Furthermore, many non-steroidal anti-inflammatory drugs are effective in the acute treatment of menstrual migraine. A previous trial demonstrated that a combination of sumatriptan 85 mg and naproxen 500 mg was more effective than placebo in treating menstrual migraine [16]. However, our data analysis indicated that this combination was not superior to sumatriptan 50 mg or 100 mg alone. Therefore, the use of triptans with NSAIDs remains a controversial issue. Moreover, a post-hoc analysis revealed that lasmiditan, a novel drug for migraine, achieved significant efficacy in 2-h pain freedom compared to placebo for perimenstrual migraine attacks [28]. This suggests that lasmiditan may also be a viable option for treating menstrual migraine.

The studies included in our review did not clearly differentiate between subtypes of menstrual migraine. Although many studies reported that they enrolled participants with MRM, they did not specify whether they counted the number of headache attacks outside the menstrual cycle. Thus, some participants with MRM might have actually had pure menstrual migraine (PMM), which only occurs during menstruation. Therefore, it is challenging to conduct a subgroup analysis between these two subtypes. Only one trial explicitly distinguished between MRM and PMM and performed a subgroup analysis, and the results showed no significant difference in drug efficacy between the two subtypes [18].

Our NMA also has some limitations that warrant consideration. Our NMA has several limitations that should be acknowledged. First, the validity of our conclusions is weakened by the fact that many treatment arms were based on only one RCT. Second, we could not assess the consistency between direct and indirect evidence sources because of the absence of evidence loops. Third, the lack of direct evidence compromised the reliability and validity of our results and limited our ability to draw conclusions about the relative effectiveness of different interventions. Fourth, some studies did not include a placebo group and only compared other drugs, which may not reflect the true efficacy of the treatment. Finally, it is important to obtain objective diagnostic evidence through prospective headache and menstrual diaries. However, many trials did not clearly report whether headache diaries were used, which may introduce recall bias, underreporting or overreporting of headache attacks among the included patients.

Our study also suggests some directions for future research on menstrual migraine. First, an accurate diagnosis is essential. The diagnosis should be confirmed by using prospective headache and menstrual diaries. A statistical model could be applied to rule out any coincidental correlation between migraine and menstruation [29]. Second, more research is required on the effectiveness of lasmiditan, eletriptan, monoclonal antibodies targeting CGRP (eptinezumab, fremanezumab, and galcanezumab) and gepants other than telcagepant for treating menstrual migraine, as there are limited trials available. Third, pure menstrual migraine and menstrually related migraine should be differentiated when feasible, as the choice of treatment may depend on the different subgroups. Lastly, side effects should be documented and reported, which will enable clinicians to make a more informed decision on the selection of drugs.

## Conclusions

Based on the results of our analysis, we found that sumatriptan 100 mg was likely to be an optimal choice for acute management of menstrual migraine, while frovatriptan 2.5 mg twice daily was likely to be an optimal choice for short-term prophylaxis. However, our study had some limitations and further research is warranted to establish the optimal treatment for menstrual migraine.

## Supplementary Information

**Additional file 1.**

**Additional file 2.**

## Data Availability

All data supporting the findings of this study are available within the paper and its Supplementary Information.

## Abbreviations

BID: Twice a day
CI: Confidence interval
ICHD-3: The International Classification of Headache Disorders 3rd edition
MRM: Menstrually related migraine
NMA: Network meta-analysis
NSAIDs: Non-steroidal anti-inflammatory drugs
OR: Odds ratio
PMM: Pure menstrual migraine
PMPs: Perimenstrual periods
PRISMA: Preferred Reporting Items for Systematic Reviews and Meta-Analyses
QD: Once a day
RCT: Randomized controlled trial
SUCRA: Surface under the cumulative ranking
