# Supplementary material for: Comparative efficacy of different treatments for menstrual migraine: a systematic review and network meta-analysis
Source: J Headache Pain. 2023 Jul 3;24(1):81. doi: 10.1186/s10194-023-01625-x (PMC10316537; doi:10.1186/s10194-023-01625-x)

**eFigure 1** Flowchart of study identification, screening and inclusion

**eFigure 2A** Risk of bias assessment for 8 studies included in the NMA.

**eFigure 2B** [Risk of bias assessment for 6 crossover trials included in the NMA.](https://www.google.com/url?q=https://www.riskofbias.info/welcome/rob-2-0-tool/rob-2-for-crossover-trials&sa=D&sntz=1&usg=AOvVaw1c4EiFhJb9cQg8jj5dPmMJ) .

**eFigure 3A** Pairwise comparison of interventions with placebo----mean percentage of PMPs without MRM


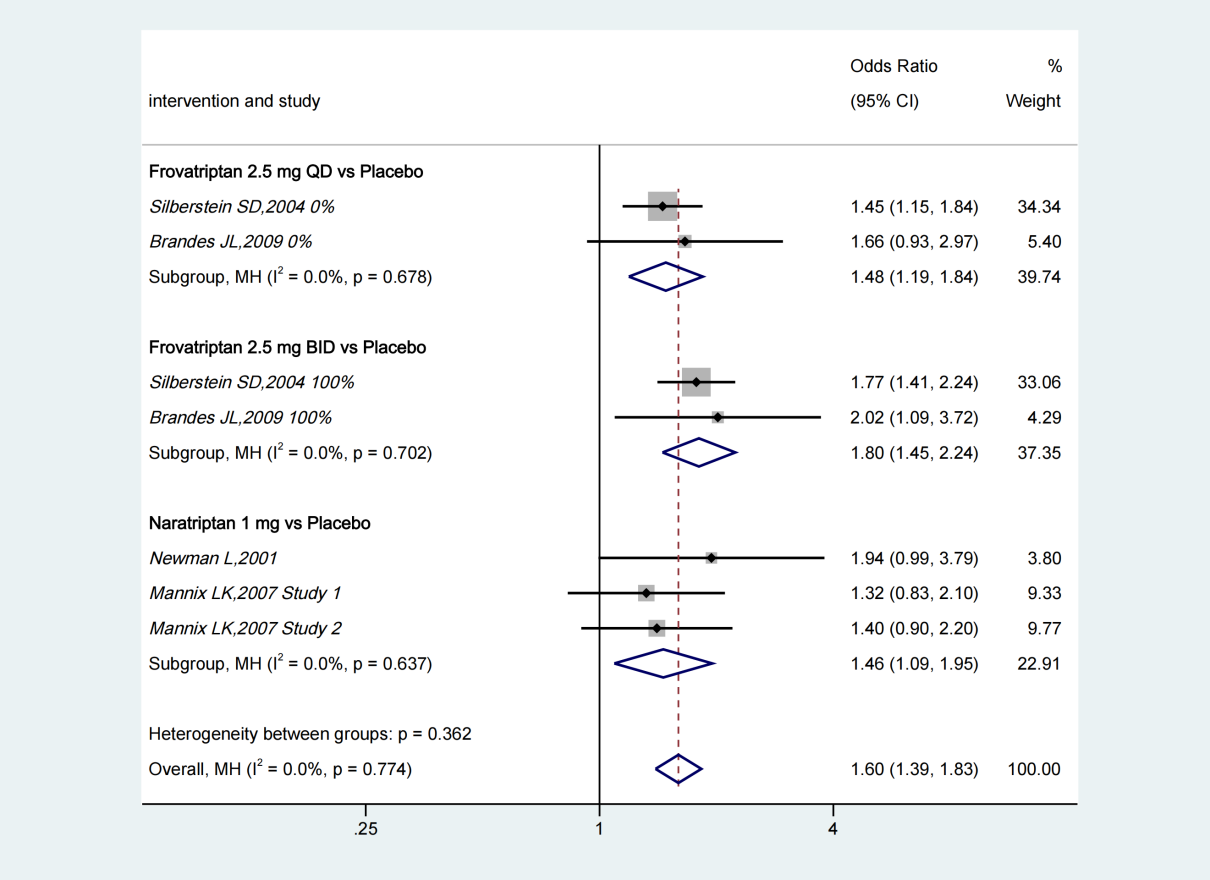


**eFigure 3B** Pairwise comparison of interventions with placebo----all adverse events


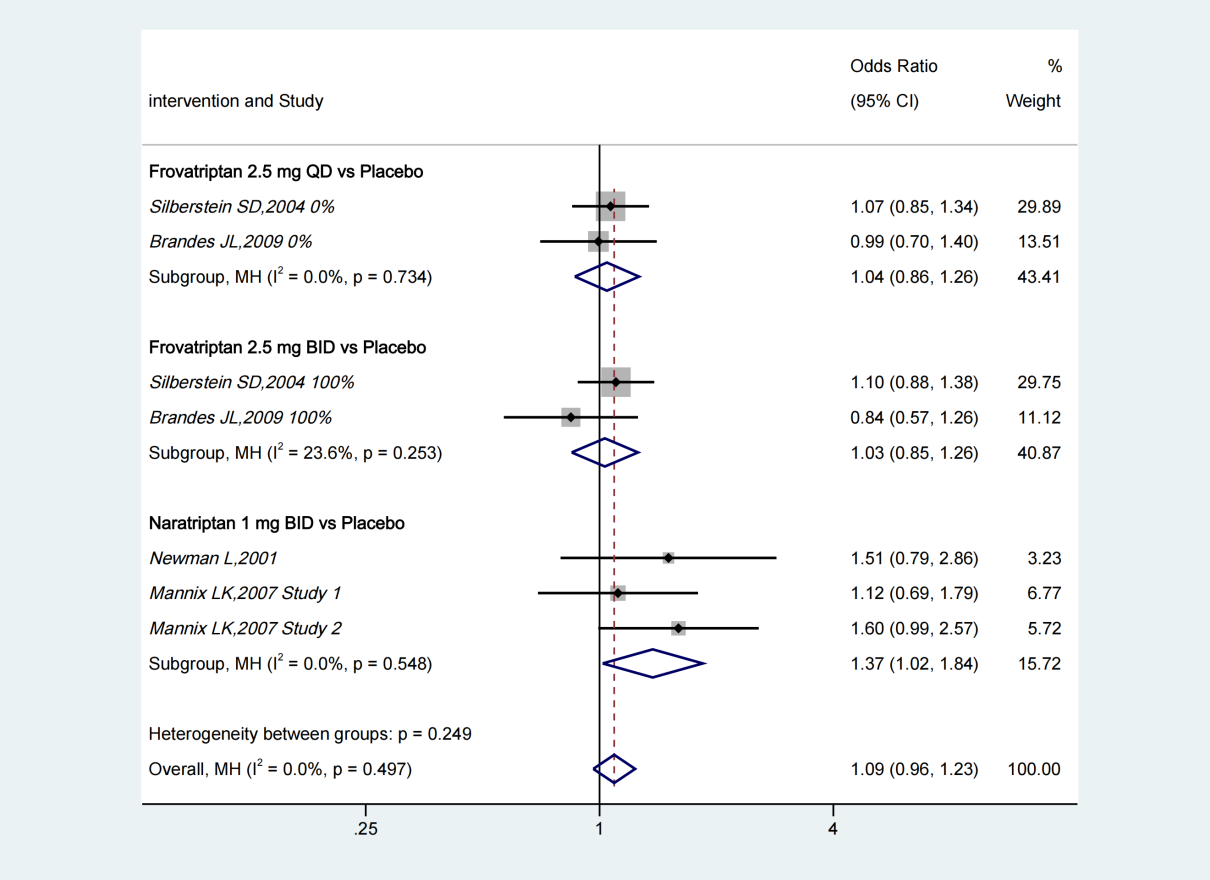


**eFigure 3C** Pairwise comparison of interventions with placebo----*2-hour pain freedom (percentage of patients)*


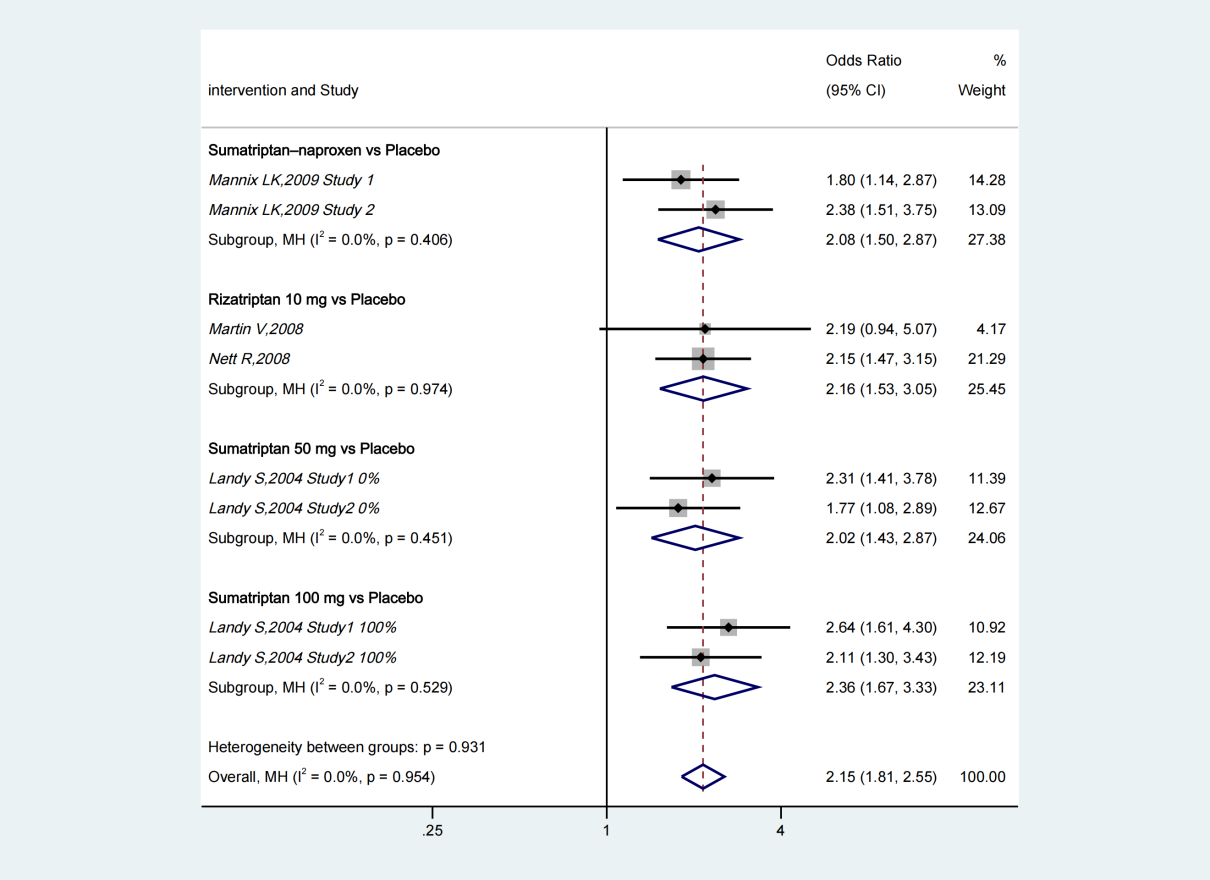


**eFigure 3D** Pairwise comparison of interventions with placebo----r*ecurrent episodes at 24 hours (percentage of attacks)*

**
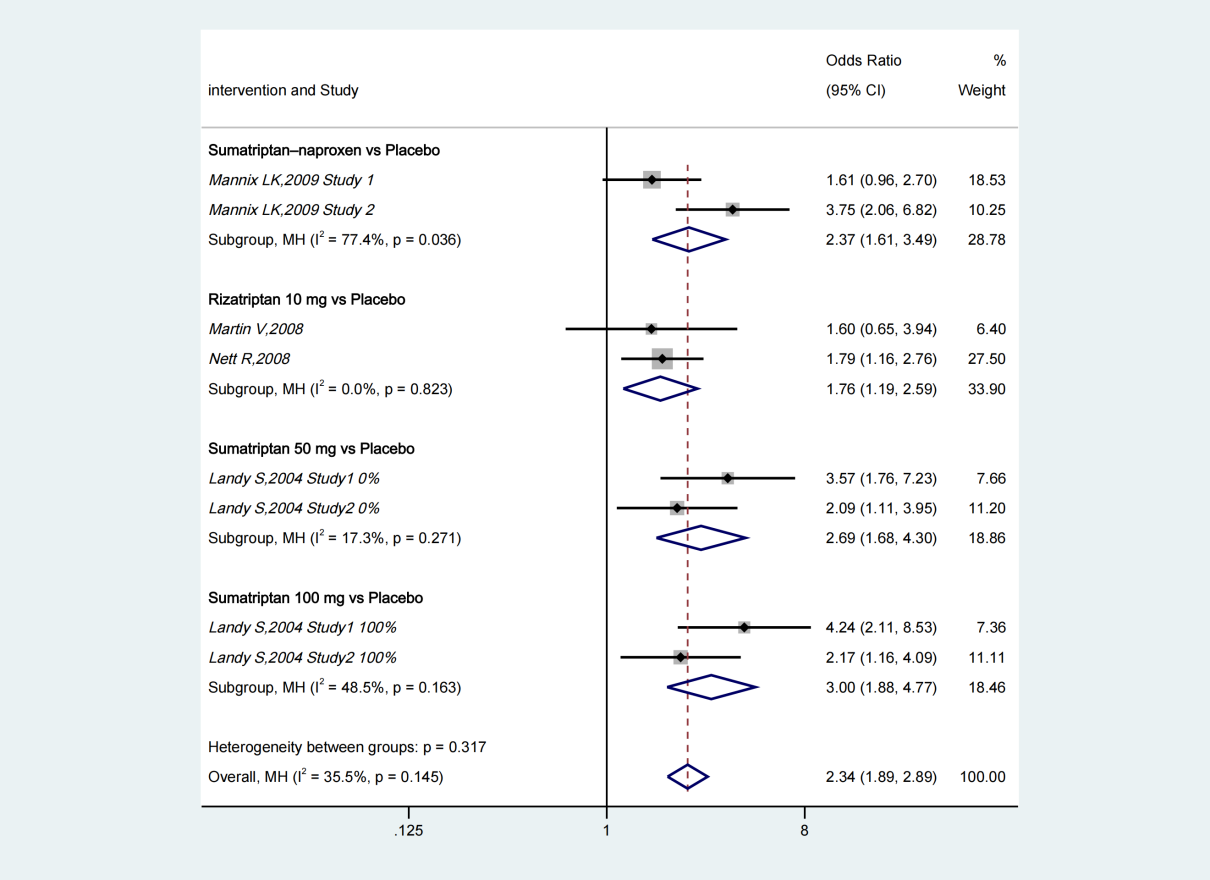
**

**eFigure 4A** Funnel plot of “ mean percentage of PMPs without MRM”

**eFigure 4B** Funnel plot of “2-hour pain freedom (percentage of patients)”

**
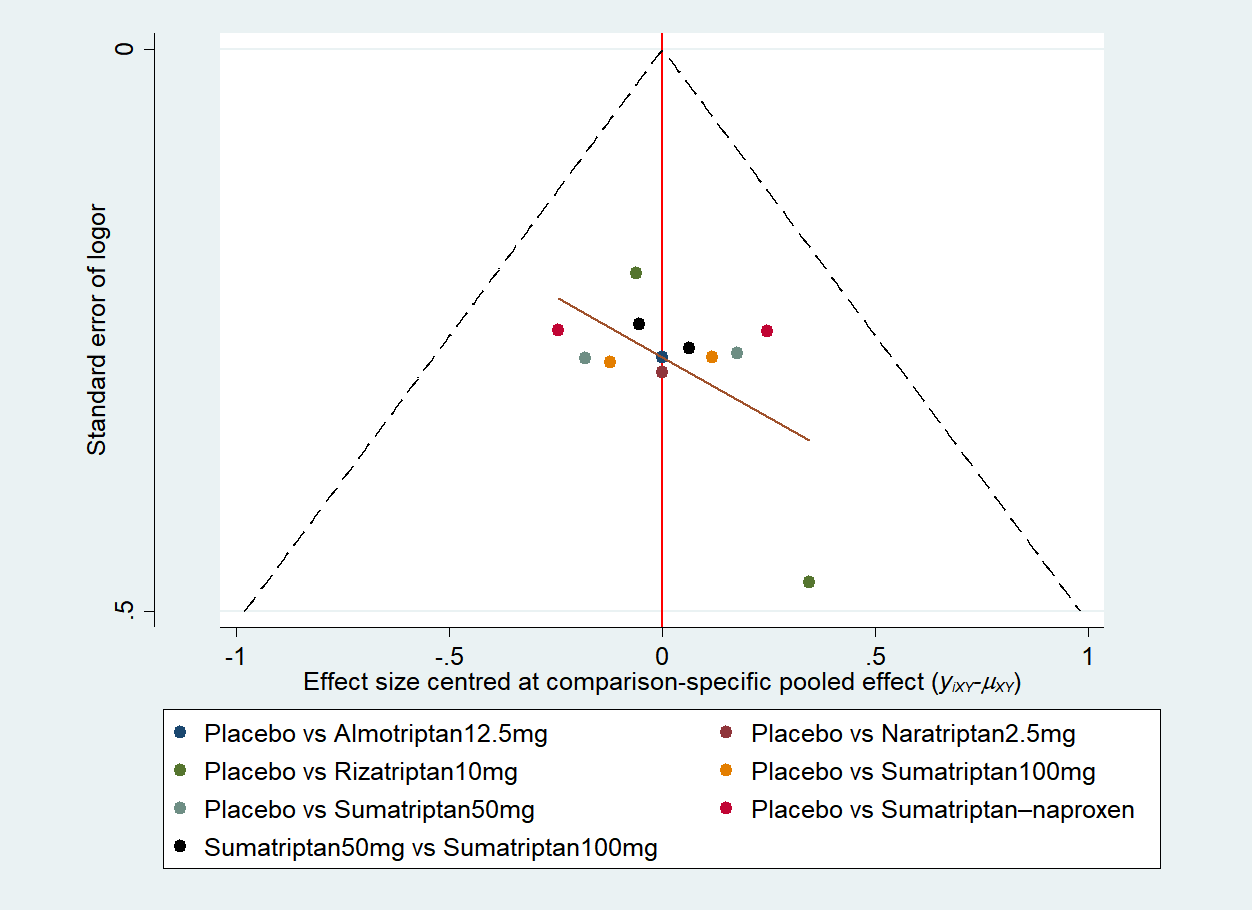
**

**eFigure 5A** network evidence plot of (1) *mean percentage of PMPs without MRM*; (2) *all adverse events*

**eFigure 5B** network evidence plot of *2-hour pain freedom (percentage of attacks)*

**eFigure 5C** network evidence plot of *recurrent episodes at 24 hours (percentage of attacks)*

**eFigure 6A** Sensitivity analysis：Forest plot of *mean percentage of PMPs without MRM*

**
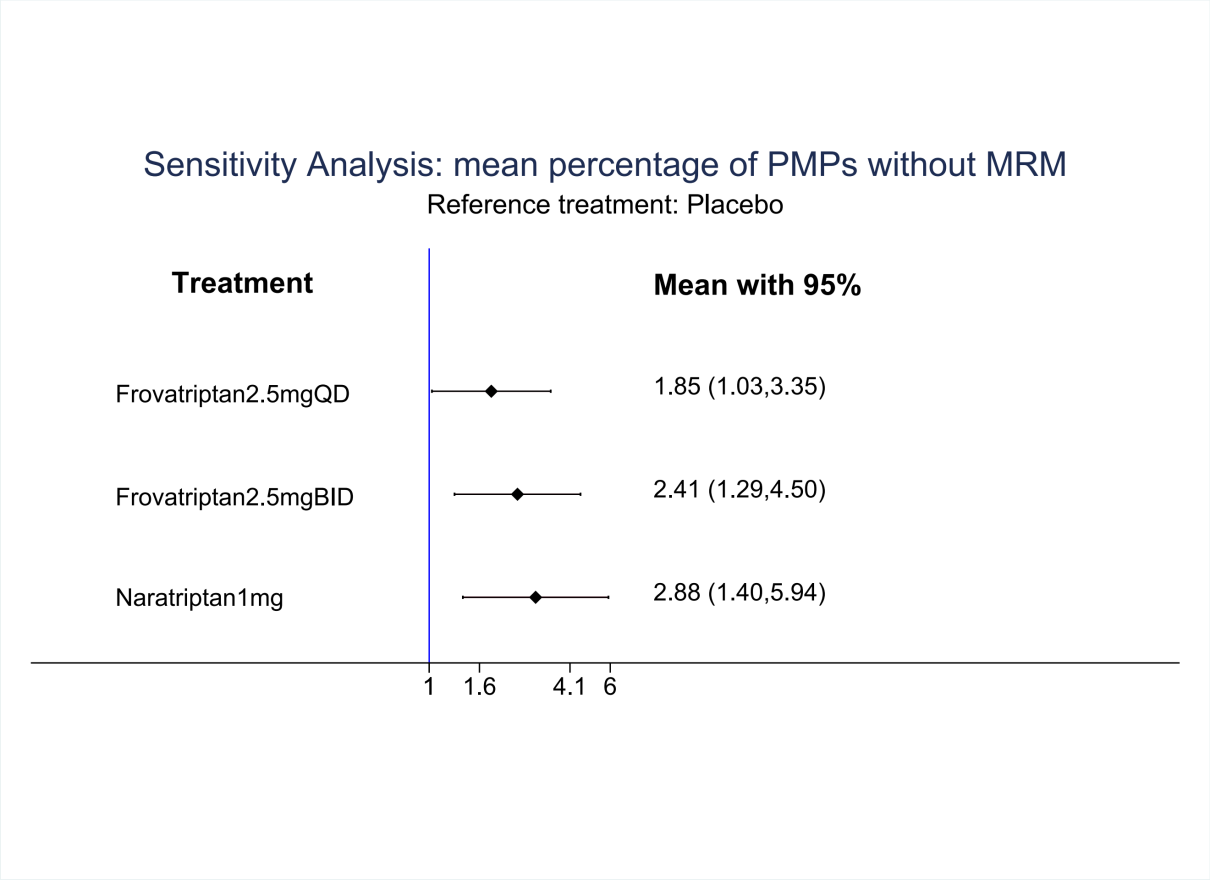
**

**eFigure 6B** Sensitivity analysis：Forest plot of *2-hour pain freedom(percentage of patients)*


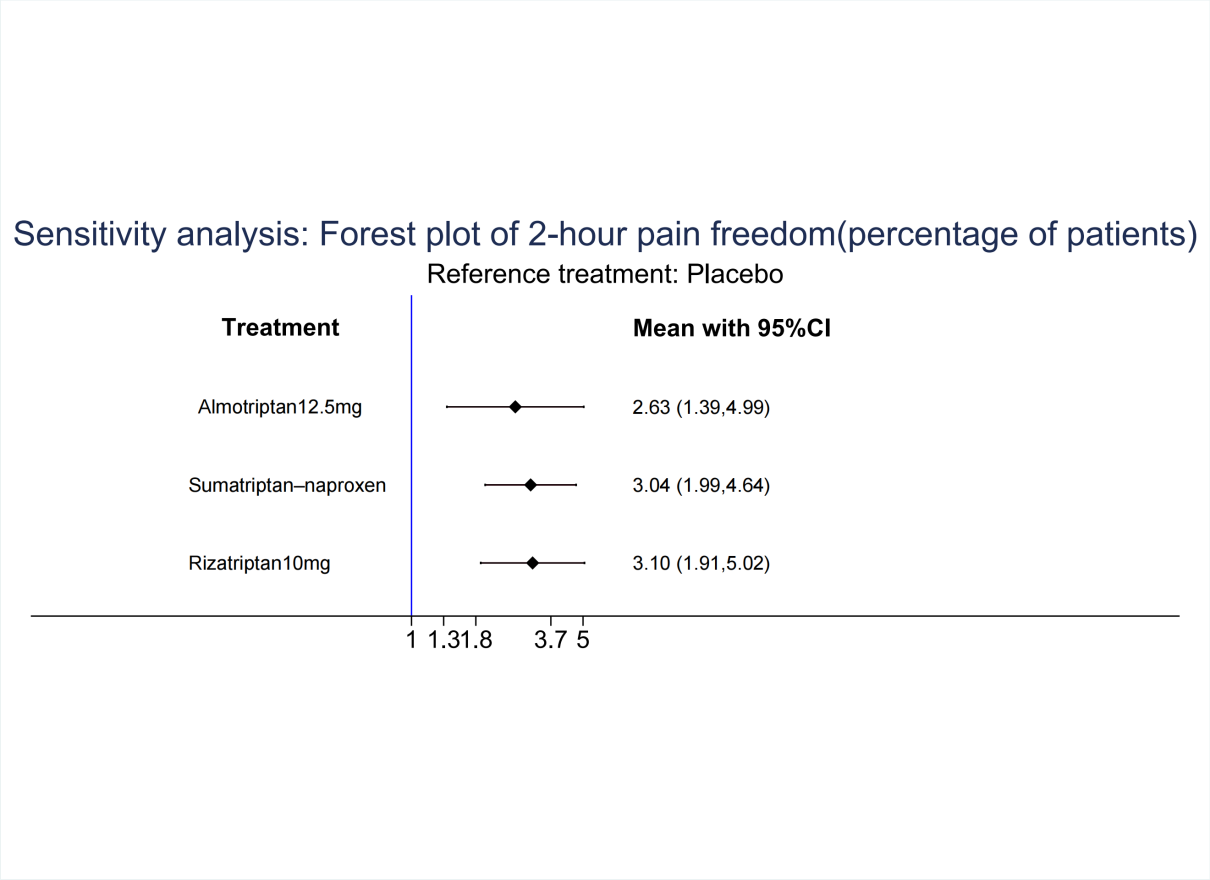

Supplement: Supplementary file 1 — Additional file 1. [file 10194_2023_1625_MOESM1_ESM.docx]
