# Supplementary material for: Comparative efficacy of different treatments for menstrual migraine: a systematic review and network meta-analysis
Source: J Headache Pain. 2023 Jul 3;24(1):81. doi: 10.1186/s10194-023-01625-x (PMC10316537; doi:10.1186/s10194-023-01625-x)
Supplement: Supplementary file 2 — Additional file 2. [file 10194_2023_1625_MOESM2_ESM.docx]

**eTable 1** Search Strategy

**eTable 2A** SUCRA of the *mean percentage of PMPs without MRM*

**eTable 2B** SUCRA of the *all adverse events*

**eTable 2C** SUCRA of the *2-hour pain freedom (percentage of patients)*

**eTable 2D** SUCRA of the *2-24 hours sustained pain freedom(percentage of patients)*

**eTable 2E** SUCRA of the *2-hour pain freedom (percentage of attacks)*

**eTable 2F** SUCRA of the *recurrent episodes at 24 hours (percentage of attacks)*

**eTable 3A** Sensitivity analysis：SUCRA of *mean percentage of PMPs without MRM*

**eTable 3B** Sensitivity analysis：SUCRA of of *2-hour pain freedom(percentage of patients)*
